# Postoperative Rehabilitation for Thoracic Disc Herniation in a Professional Rugby Player: A Case Report

**DOI:** 10.7759/cureus.30423

**Published:** 2022-10-18

**Authors:** Tanaka Kohei

**Affiliations:** 1 Rehabilitation Medicine, Osaka Police Hospital, Osaka, JPN

**Keywords:** physical therapy rehabilitation, rehabilitation program, post-operative recovery, return to sport, disc hernia

## Abstract

Thoracic disc herniation (TDH) is a very rare condition compared to cervical and lumbar disc herniation. Patients commonly attend rehabilitation programs after surgery, and the beneficial effects of rehabilitation for cervical and lumbar disc herniation have been reported. However, a postoperative rehabilitation program for patients with TDH has not yet been reported. This case report describes a postoperative rehabilitation program and chronological changes in physical function after surgery for TDH in a professional rugby player. We report the case of a 31-year-old male professional rugby player diagnosed with TDH at T1-T3 and ossification of the yellow ligament at T2-T3. It was difficult for the patient to walk because of the severe spasticity in the lower extremities. The patient underwent surgery to remove the ossified yellow ligament at T2-T3 and posterior thoracic interbody fusion (PTIF) at T1-T3. Rehabilitation programs such as joint mobilization and stability training were initiated after surgery. Spasticity gradually decreased, and the patient could walk unassisted three weeks after surgery and return to field training four months after surgery. This case report suggests that a postoperative rehabilitation program could be safely provided to patients with TDH in the early postoperative period, which may be effective in improving physical function.

## Introduction

Disc herniation is a fairly common condition. However, thoracic disc herniation (TDH) is a very rare condition compared to cervical and lumbar disc herniation [1]. The frequency of TDH is estimated to be approximately one patient per million people [2]. Most TDHs are located below the T7-T8 disc and mainly at T11-T12 [3]. The characteristic feature of TDH is a high frequency of calcification and ossification. The most common clinical sign is pain (intercostal neuralgia, back pain), which may be accompanied by ataxia on walking, progressive impairment of the lower extremity function with pyramidal tract syndrome, or bladder sphincter signs. These symptoms are caused by sensory or motor deficits associated with slow medullary compression. It can also occur after trauma and rapidly develop neurological signs. A history of trauma is found in 3%-37% of TDH [4]. Indications for surgery are present when functional symptoms do not respond to medical treatment and/or neurological symptoms appear or worsen. Although several surgical approaches are available, the posterolateral approach is indicated for soft lateral hernias or when there are multiple levels of compression due to the ossification of the posterior longitudinal ligament. Posterior approaches with arthrectomy extending to the pedicle almost always require instrumented fusion. Surgical treatment of TDH has been reported to result in neurological improvement in 53% of patients, stability in 42%, and worsening in 5% of patients [5].

 Rehabilitation programs, such as exercise therapy and advice to return to normal activities by a physical therapist, are commonly applied after surgery. Although the quality of evidence is low, postoperative rehabilitation programs for cervical and lumbar disc herniation seem to lead to a faster decrease in pain and disability [6-7]. While several cases of postoperative TDH have been reported [8-9], progress in physical function and rehabilitation programs for postoperative patients with TDH has not been reported. In this case report, we describe a postoperative rehabilitation program and chronological changes in physical function after posterior thoracic interbody fusion (PTIF) for TDH in a professional rugby player.

## Case presentation

This study was approved by the ethics committee of the Osaka Police Hospital (approval number:1523). Informed consent was obtained from the patient.

**Figure 1:**
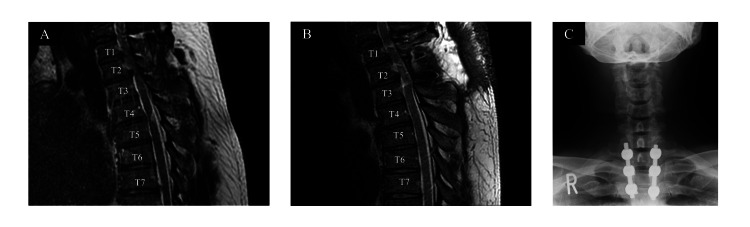
The images of MRI and X-ray for cervical and thoracic spine. A, MRI image of pre-operation; B, MRI image of post-operation; C, X-ray image of post-operation

We report the case of a 31-year-old male professional rugby player diagnosed with TDH at T1-T3 and ossification of the yellow ligament at T2-T3 (Figure 1). The patient noticed slight muscle weakness in the lower extremities approximately a month before surgery and had difficulty exercising a week later. Although the progression of muscle weakness was not severe, spasticity in the lower extremities gradually increased, and the patient had difficulty with ambulation. The patient underwent surgery to remove the ossified yellow ligament at T2-T3 and PTIF at T1-T3. During surgery, the caudal side of the T1 spinous process and T2 spinous process were resected. The rehabilitation program was initiated on the first postoperative day. Since the patient could walk without assistance, he was discharged home three weeks after the surgery and continued outpatient physical therapy. After discharge from the hospital, the patient started exercise therapy at his team's training facility.

Examination of physical function

The chronological changes in physical function are presented in Table 1. A physical therapist assessed physical function. Pain severity was assessed by an interview using the numerical rating scale with a score of 0-10. The patellar tendon reflex was assessed as an indicator of spasticity of the lower extremities. The degree of neck and hip joint movement and finger floor distance were measured as indicators of flexibility. Lower limb muscle strength was assessed using manual muscle testing (MMT). Gait speed was measured using a 10 m straight course at comfortable or maximum speed. Gait speed was measured twice, and the maximum speed was used as the representative value. Once the patient was able to run, we measured the running speed using a 10 m straight course. The standing long jump and single-leg hop tests were measured as indicators of agility. The standing long jump was measured by standing immediately behind a line with feet shoulder-width apart, and the participant jumped as far as possible with their feet together [10]. The single-leg hop test was performed by jumping as far as possible with one leg while standing immediately behind the line [11]. If a patient loses balance or falls during these tests, the trial is considered invalid and must be repeated.

**Table 1:** The chronological changes in physical function. NRS, numerical rating scale

		Pre-operation	Post-operation					
			2 weeks	1 month	2 months	3 months	4 months	5 months
Leg pain (NRS)		2	2	2	1	0	0	0
Patellar tendon reflex		Hyper/hyper	Hyper/hyper	Hyper/hyper	Hyper/hyper	Normal/hyper	Normal/normal	Normal/normal
Tandem Romberg test		Positive	Positive	Negative	Negative	Negative	Negative	Negative
Range of motion (degree)								
Neck	Flex/extension	40/50	30/45	35/50	40/50	40/50	45/50	45/50
	Lateral bending	45/25	40/30	40/30	40/30	40/30	40/30	40/30
	Rotation	45/75	45/80	45/80	50/80	50/75	55/80	60/80
Straight leg raise (degree)		55/60	60/60	70/65	70/65	70/70	80/80	80/80
Floor finger distance (cm)		10	18	10	11	7	8	6
Manual muscle testing								
Hip	Flexion	4/4	4/5	5/5	5/5	5/5	5/5	5/5
	Abduction	4/4	4/5	5/5	5/5	5/5	5/5	5/5
Comfortable gait speed (m/s)		1.08	0.92	1.12	1.33	1.53	1.46	1.42
Maximum gait speed (m/s)		1.28	1.00	1.35	1.52	1.79	1.62	1.74
Running speed (m/s)				2.35	2.44	2.96	330	3.17
Standing long jump (cm)							100	190
Single leg hop distance (cm)								100/95

Chronological changes in physical function

During the postoperative days, the patient required assistance with a walker for walking as he still had the same level of spasticity as before the surgery. On postoperative day four, muscle weakness was observed in the right lower extremity. MRI revealed that the patient had edema around the surgical site, and steroids were administered intravenously for three days and internally for two weeks. The edema decreased after two weeks, as confirmed by MRI, and the muscle weakness in the right lower extremity also improved. Lower limb muscle strength improved to the fifth level of MMT within one month after surgery. The patellar tendon reflex was extremely hyperactive due to increased spasticity, which gradually decreased two months after surgery. Similarly, stiffness in the lower limbs was reduced, and flexibility gradually improved. As spasticity decreased, coordinated movement of the lower limbs improved, and agility training, such as quick steps and jumps, was started carefully six weeks after surgery.

Rehabilitation program

**Figure 2:**
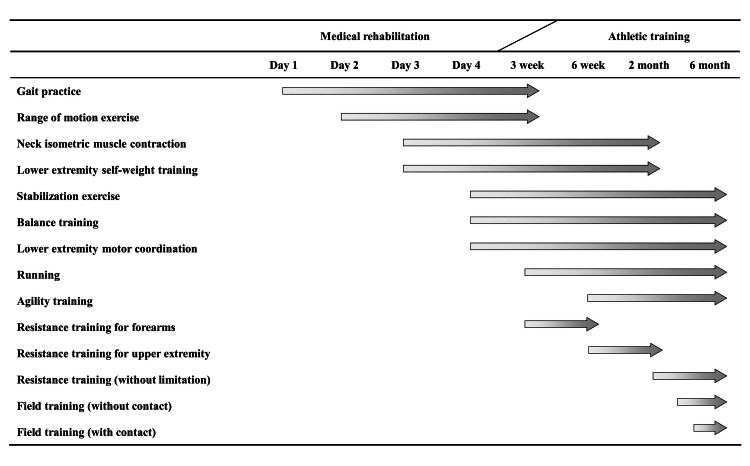
The time course of postoperative rehabilitation program.

A physical therapist initiated exercise therapy on the first postoperative day. The time course of the postoperative rehabilitation program is shown in Figure 2. Exercise therapy was conducted once a day for three weeks in the hospital and continued twice a week after discharge for five months after surgery. The program started with ambulation practice. Passive range of motion (ROM) exercises of the neck and extremities were initiated on the second postoperative day. We added active ROM exercises and stretching while confirming no exacerbation of pain or presence of neurological symptoms during the exercise. From the third postoperative day, cervical spine and trunk stabilization exercises were started without joint movement (e.g., local muscle contraction by drawing in). From postoperative day four, bridge and plank exercises were added as trunk stabilization exercises, and the patient's posture was assessed by a physical therapist. The exercise load gradually increased with a combination of multiple joint movements. Balance training and lower-limb motor coordination training were also started on postoperative day four. Running training started three weeks after surgery. Upper limb muscle training started with forearm resistance training, such as arm curls, in consideration of the stress on the surgical site. While confirming that there was no exacerbation of pain or any neurological symptom, resistance training of the muscles around the neck and shoulder joint was initiated using Theraband. Under the supervision of the athletic trainers of his team, the patient gradually increased the resistance training load using machines two months after the surgery. The patient returned to field training four months after surgery.

## Discussion

We described the progress of physical function and postoperative physical therapy in a rugby player who had undergone PTIF for TDH with ossification of the yellow ligament. The most serious problem, in this case, was a walking disability due to spasticity in both lower limbs. Spasticity in both lower limbs, which had occurred preoperatively, did not improve even several weeks after surgery, and motor coordination was severely impaired. Exercise therapy is reported to be effective in decreasing spasticity [12-13], and we repeatedly performed low-difficulty exercises, such as flexion and extension of the lower limbs on the bed and ball control with the plantar feet in the sitting position, as motor coordination training. With the decrease in spasticity, motor coordination improved, and the patient gradually became able to perform exercises in the standing position. However, with hyperspasticity, it is difficult to perform the exercise because of the stretch reflex that occurs during rapid movements.

 As TDH is a very rare disease, there are no established protocols for postoperative physical therapy. Postoperative physical therapy after cervical or lumbar disc herniation may include ROM and stabilization exercises during the early postoperative period [6, 14-15]. After surgery, ROM exercise is an important intervention because of the risks of postoperative neck pain and cervical ROM limitation due to spinal immobilization [16]. However, excessive joint mobilization in the early period after spinal fusion surgery should be carefully managed because bone fusion is insufficient, and implant loosening can occur. In addition, edema and hematoma are sometimes observed around the surgical site in the early postoperative period, and it is necessary to avoid applying excessive stress to the surgical site. Therefore, ROM exercises and stretching were started with mild intensity and gradually increased while assessing the pain associated with movements and improving the smoothness of motion. After spinal surgery, spinal stability tends to be reduced because of weakness in the trunk muscles [17-18]. Additionally, patients with herniation were reported to have decreased trunk muscle strength compared to healthy individuals [19]. Spinal instability may increase intervertebral discs and facet joint stress, leading to herniation or spondylolysis. Therefore, in this case, spinal stabilization exercises were initiated early in the postoperative period. Considering the stress on the surgical site, the exercise was started from a posture with the spine fixed in a neutral position, and the difficulty level was gradually adjusted to include limb and spine movements.

## Conclusions

In this case, physical therapy was initiated in the early postoperative period to improve physical function. No adverse events due to exercise were observed, and physical function improved to enable field training at five months post-operation. This case report suggests that physical therapists can safely provide exercise therapy for patients who undergo PTIF for TDH in the early postoperative period, which may be effective in enhancing the recovery of postoperative physical function.
